# A Mutant *Brassica napus* (Canola) Population for the Identification of New Genetic Diversity via TILLING and Next Generation Sequencing

**DOI:** 10.1371/journal.pone.0084303

**Published:** 2013-12-20

**Authors:** Erin J. Gilchrist, Christine H. D. Sidebottom, Chu Shin Koh, Tanya MacInnes, Andrew G. Sharpe, George W. Haughn

**Affiliations:** 1 Department of Botany, University of British Columbia, Vancouver, British Columbia, Canada; 2 National Research Council Canada, Saskatoon, Saskatchewan, Canada; University Paris South, France

## Abstract

We have generated a *Brassica napus* (canola) population of 3,158 EMS-mutagenised lines and used TILLING to demonstrate that the population has a high enough mutation density that it will be useful for identification of mutations in genes of interest in this important crop species. TILLING is a reverse genetics technique that has been successfully used in many plant and animal species. Classical TILLING involves the generation of a mutagenised population, followed by screening of DNA samples using a mismatch-specific endonuclease that cleaves only those PCR products that carry a mutation. Polyacrylamide gel detection is then used to visualise the mutations in any gene of interest. We have used this TILLING technique to identify 432 unique mutations in 26 different genes in *B. napus* (canola cv. *DH12075*). This reflects a mutation density ranging from 1/56 kb to 1/308 kb (depending on the locus) with an average of 1/109 kb. We have also successfully verified the utility of next generation sequencing technology as a powerful approach for the identification of rare mutations in a population of plants, even in polyploid species such as *B. napus*. Most of the mutants we have identified are publically available.

## Introduction

For generations, humans have been breeding and cultivating plants to take advantage of the genetic variation that has arisen over time and is distributed throughout the plant’s genome. Forward genetics has been used to identify the roles of many genes involved in plant growth and development. However, with the advent of genome sequencing, we are now aware that the function of most genes is unknown and can only be predicted from sequence similarity or expression patterns. For this reason, several reverse genetic techniques have been developed that enable researchers to identify plants with mutations in genes of known sequence. The characteristics of a plant carrying specific mutations can then be studied to see if changes in the gene of interest affect any phenotypic traits. 

Targeting Induced Local Lesions in Genomes (TILLING) is a reverse genetics technique that allows direct, cost-efficient screening for point mutations or single nucleotide polymorphisms (SNPs) in a specific gene of interest within an acceptable time frame for most projects. TILLING can be used to screen for natural genetic variation in wild populations, or for induced polymorphisms in mutagenised populations. Chemical mutagenesis using ethylmethane sulphonate (EMS) or ethyl nitrosourea (ENU) has been long used to generate SNPs in the DNA sequence of many plant species for both applied and basic research. Mutations induced in this manner are distributed randomly throughout the genome and at a high enough frequency that examinations of gene function on a genomic level is possible [1]. They can result in a variety of loss-of-function or, more rarely, gain-of-function phenotypes. Loss-of-function alleles may eliminate gene function completely through nonsense or splice junction mutations, but more often are the result of missense alleles that result in partial loss of activity and can provide a range of alleles of different severity for any given gene. Point mutations causing dominant gain-of-function phenotypes, while rare, have also been described. One example of such a mutation has been shown to affect the ethylene response pathway (reviewed in 2). Dominant mutations have also been important in elucidating other areas of plant development such as leaf polarity (reviewed in 3) and host-pathogen interaction (reviewed in 4). 

One method of TILLING makes use of the technique described by Colbert et al. [5], using a mismatch-specific endonuclease for identifying SNPs in genes of interest. In this procedure, DNA from several different lines is pooled and amplified using gene-specific primers. The PCR products (amplicons) are denatured and allowed to randomly re-anneal before being digested with an endonuclease that recognises mismatches in the paired DNA molecules. Following renaturation, mismatches (heteroduplexes) in the amplified DNA occur if a pool of DNA includes at least one plant with a mutation in the amplified region. Endonuclease digestion of DNA heteroduplexes results in novel DNA fragments that can be detected by high-performance liquid chromatography, gel electrophoresis, capillary separation, or high resolution melting curve analysis [5–9]. An alternative way of detecting rare SNPs is by directly using next generation sequencing (NGS) technologies to sequence specific genes of interest in a population of plants using multidimensional pooling strategies. We suggest using the term Sequencing Candidate Amplicons in Multiple Parallel Reactions (SCAMPRing) to describe this strategy that has been used by us and others. SCAMPRing can identify all of the mutations in that population without the need for enzymatic digestion or gel electrophoresis (for review see 10). NGS technologies can provide accurate and rapid detection of rare mutations in target genes through sequencing of specific amplified regions [11–15] or of the whole or parts of genomes of a subset of individuals within the population to be characterized [16,17]. 

TILLING is rare among reverse genetic techniques in that it can be used to identify genetic variability in either mutagenised or natural populations. Because this variation does not involve transformation of exogenous genetic material, TILLING can also be used in plant research and breeding programs in species where there are barriers to creating or marketing genetically modified varieties. 

### *Brassica napus*

*Brassica napus* (canola/rapeseed) is an important oilseed crop worldwide. The oil found in *B. napus* seeds is used not only for food and fuel, but also in cosmetics, inks, pesticides, lubricants, and coolants. The different fatty acid components of specific oils from different plants make them best suited to particular uses. For example, using selection and breeding, low erucic acid and low glucosinolate (double low or “00”) content in seeds was developed in a *B. napus* line which led to the development of the “canola” variety of this crop [18]. Canola produces seeds that are used to generate excellent edible oil that is lower in saturated fat and higher in omega-3 fatty acids than most other commercially available oils. These attributes have been shown to have a significant positive impact on human health, reducing diseases such as cancer, heart disease and some neurological disorders [19,20]. Modification of the levels and types of fatty acids in seed oil is genetically controlled, but presently, most important genetic manipulations in plants are performed using transgenic technologies that insert exogenous genes into plants that have already been conventionally bred to produce high-yields. TILLING can provide additional genetic variation required for crop improvement without using transgenes. It is adaptable to any species for which a population of mutagenised or natural variants exists. In addition, it is one of the few techniques that is suitable for use in polyploid species (such as *B. napus*). 

Forward and reverse genetics in polyploid species is complicated by the fact that each gene is present in multiple copies. Thus, a mutation in a single locus is not likely to be identified in a forward genetics screen because the loss of function of that gene will likely be masked by the activity of the homeologous gene(s). Reverse genetics, therefore, is a more practical approach to functional analysis of gene function in polyploids. Through TILLING, mutations in individual homeologous genes can be identified independently and then introgressed into the same line in order to observe the knockout phenotype and identify the function of this gene *in planta*. This same approach can be applied to address redundancy problems for genetics in diploids since each homologue of a multigene family can be targeted independently and combined into a single line using genetic crosses. 

A necessary prerequisite for TILLING in *B. napus* is a population with significant genetic variation. Such a population (either mutagenised or natural) can be used to screen for variation in genes with the potential to improve agronomically important traits affecting, for example, meal for food and animal feed, and diversified seed oil content for nutritional and industrial uses. Here we report the construction of a population of more than 3,000 mutagenized lines of *Brassica napus* (canola cv. *DH12075*) with a mutation density suitable for TILLING. This population, in addition to others published previously [15,21–23] adds to the growing list of reverse genetic resources available for *Brassica* species. In addition, we have used this population to compare the classical TILLING technique with NGS technology as cost-effective and efficient methods for detecting point mutations in genes of known sequence. 

## Materials and Methods

### Mutagenesis

Seeds from *B. napus* (canola cv. *DH12075*) were divided into 4 g aliquots. (approximately 1,000 seeds) and surface sterilized for ten min in a 250 ml flask in 10% bleach solution followed by rinsing 4x in dH_2_O. After drying briefly on a paper towel, seeds were transferred to a fresh 250 ml flask and 50 ml of the appropriate concentration of EMS solution (0.25%, 0.3% or 0.4% in distilled water) was added. The flask was placed on an orbital shaker and shaken gently for between 14 and 16 hours. Seeds were then washed by pouring through a funnel lined with cheesecloth or Miracloth (EMD Millipore, Billerica, MA, USA) so that the EMS solution was collected into a 1L flask containing 2x sodium thiosulfate (15.8 g in 500 ml H_2_O) for neutralisation. Seeds were rinsed 2x with fresh sodium thiosulfate (15.8 g in 1L H_2_O) and 2x with distilled water. After drying briefly on a paper towel, seeds were spread evenly onto two layers of Whatman paper dampened with water in 4, 15 cm petri dishes and sealed with Parafilm®M (Pecheney Plastics Packaging, Chicago, IL, USA) to prevent drying. Seeds were allowed to germinate at room temperature for two to three days in the dark before planting directly in soil.

### Planting and growth

Plants that germinated from mutagenized seeds (the M_1_ generation) within 3 to 10 days were transferred to 40 mm square pots filled with soil and grown in a greenhouse. Plants were bottom watered with 15-5-15 fertiliser. When plants began to flower, plastic pollination bags (12 x 24 inch, microperforated, 0.75 gauge BOPP, Chantler Packaging Inc., Mississauga, ON, Canada) were placed over each plant. The bagged plants were shaken daily to promote self-pollination within the bag. Bags were removed and seeds harvested when several siliques were brown and dry (four to six months). These M_2_ seeds from the mutagenized M_1_ plants were planted in 40 mm pots (6 seeds from each M_1_ parent per pot). If more than one seed germinated, the remaining plants were discarded so that each M_2_ line represented a single M_1_ parent. M_2_ plants were grown until flowering, at which point leaves were taken from each plant and frozen in labelled plastic bags at ^-^80 °C for DNA extraction at a later date (see below). Plants were allowed to grow until they had produced a minimum of 100 seeds. Plants unable to produce 100 seed after four to eight months were discarded. Seeds were dried using an Excalibur food dehydrator (Excalibur, Fort Lauderdale, FL, USA), and stored in glassine bags over t.h.e.* Desiccant (EMD Millipore, Billerica, MA) at 5% relative humidity in plastic boxes (Rubbermaid) at room temperature.

### DNA extraction and pooling

Leaf tissue collected from each M_2_ plant at the time of flowering and stored in plastic bags at ^-^80 °C was used for DNA extraction. If the plant yielded approximately 100 seeds or more, DNA was then extracted for TILLING from the stored M_2_ leaves. 1 mg of leaf tissue was ground in 1.5 ml tubes using a Neiko 8" Mini Drill Press (Neiko, USA) with a Konte pestle (Kimble Chase Life Science, Vineland, NJ, USA) attached or with a Precellys®24 tissue bead homogenizer (Bertin Technologies Corp., Rockville, MD USA) using 0.5 mm zirconium oxide beads (Next Advance Inc., Averill Park, NY, USA). DNA was then extracted using Plant DNAzol (Invitrogen Canada Inc. Burlington, ON, Canada), treated with RNAase as directed and dissolved in 0.2 ml TE (10 mM Tris, 1 mM EDTA pH 7.4), before quantification using a NanoDrop 1000 spectrophotometer (Thermo Fisher Scientific, Wilmington, DE USA) After quantification, DNA was diluted to 1 ng/ul in 1 ml 10 mM Tris, 1 mM EDTA pH 7.4 and arrayed in 96-well deep well plates. The diluted DNA was later pooled 4-fold to generate both row and column pools for TILLING.

### Primer design and PCR Amplification

Primers were designed to amplify a fragment between 1,300 bp and 1,800 bp in size depending on available sequence data and amplification conditions. Primers were designed to amplify a single locus and tested to ensure that only one of the homeologous loci was amplified before TILLING. Primers were purchased from MWG Biotech, Inc. (High Point, NC, USA), diluted to a concentration of 100 uM in 10 mM Tris, 1 mM EDTA pH 7.4 and used at a final concentration of 0.2 mM in a mixture of 3:2 (labeled:unlabeled) for the forward (IRD700-labeled) primers and 4:1 (labeled:unlabeled) for the reverse (IRD800-labeled) primers as per Colbert et al. [5]. PCR was also performed according to Colbert et al. [5]: 10 ul PCR reactions with 2.5 ng–5 ng of genomic DNA were used for amplification in 96-well or 384-well PCR plates using ExTaq polymerase (Takara Bio Inc., Japan), but with 0.6 times the recommended concentration of ExTaq buffer and 2 mM MgCl_2_. PCR cycles were as follows: 95 °C for 2 min; eight cycles of [94 °C for 20 sec, 73 °C for 30 sec (decrementing 1 °C per cycle), 72 °C for 1 min]; 45 cycles of: [94 °C for 20 sec, 65 °C for 30 sec, and 72 °C for 1 min]; 72 °C for 5 min; 99 °C for 10 min (denaturation and inactivation of Taq enzyme); and 70 cycles of 20 sec at 70 °C (decrementing 0.3 °C per cycle for random reannealing to allow hybridisation of mutant and wild-type molecules), hold at 4 °C.

### Preparation of celery juice extract

Crude celery juice extract (CJE) was prepared as described by Till et al. [24]. Briefly, 0.5 kg of celery was processed in a kitchen-quality juicer until liquefied. Tris HCl (pH 7.7) was added to 0.1 M along with Phenylmethylsulphonylfluoride (PMSF) to 100 mM. The solution was spun at 2,600 g for 20 min and the supernatant removed, then brought to 25% saturation in (NH_4_)_2_SO_4_, before being mixed for 30 min at 4 °C, and spun at 15,000 g for 40 min at 4 °C. The supernatant was removed again and adjusted to 80% saturation in (NH_4_)_2_SO_4_, mixed for 30 min at 4 °C, and spun at 15,000 g for 1.5 h at 4 °C. The pellet from this cut was resuspended in 1/10 the starting volume of 0.1 M Tris HCl (pH 7.7), 100 mM PMSF. The suspension was dialysed against 8 L of the same buffer, four times, for one hour each time at 4 °C using Spectrapore dialysis tubing (10,000 MW cut-off). Aliquots were stored at ^-^80 °C and were spun at approximately 2,000 g for one min before use to remove any tissue debris.

### CJE digestion, sequence analysis and identification of mutants

PCR products were digested with CJE by adding 20 μl of 1.5x CJE in buffer (100 mM MgSO4, 100 mM HEPES, 300 mM KCl, 0.02% Triton X-100, 0.002 mg/ml BSA, and 0.2% to 0.3% crude CJE) directly to the PCR reactions and incubating at 45 °C for 15 min. Reactions were stopped by adding 2.5 μl of 0.5 M EDTA. The DNA was purified by passage through G50 Sephadex in 96-well Millipore Multiscreen® filtration plates (Millipore Corporation, Billerica, MA) and concentrated for 30 min at 90 °C before running on a 25 cm long Li-cor acrylamide gel with a 0.4 mm wide, 96-well sharkstooth comb. Analysis of the gel images was done using GelBuddy [25] to define lanes and estimate sizes of cleavage products. The correlation of row and pool columns indicated which individual plant carried the mutation. Most mutations were sequenced in both directions using either the same forward or reverse primers as for PCR or an internal primer designed for sequencing. Sequence analysis was performed using Sequencher 4.2 (Gene Codes Corporation, Ann Arbor, MI, USA) and the potential effect of the mutations was predicted using PARSESNP [26].

### Illumina sequencing of candidate amplicons

The approach we used to test the use of NGS technologies to screen for mutations involved multi-dimensional pooling of targeted PCR amplicons generated from extracted genomic DNA of a set of 384 lines from the mutant population. The locus used for testing this technique was: bn1 (BnSAD: stearoyl-acyl carrier protein desaturase, partial gene). Overlapping amplicons were generated to cover this target gene, using the same PCR conditions described above. They were then pooled and different oligomer tags were added to each DNA pool for barcoding purposes to allow for sufficient resolution of individual mutant lines. The pooling strategy enabled resolution of mutations to one in six lines within the 384 lines arrayed (Figures S1 and S2). Amplicons were generated from sets of column, row and plate pools comprised of 96 lines, before shearing (Covaris, Inc. Woburn, Massachusetts, USA) and the addition of Illumina barcode adapters (Illumina Inc., San Diego, CA, USA). These were then pooled further for sequencing on a single lane of a Genome Analyzer IIx instrument (Illumina Inc., San Diego, CA, USA), providing 100 bp paired reads post-run processing, enabling sequencing reads to be partitioned according to the barcodes (Cassava 1.8, Illumina Inc., San Diego, CA, USA). All raw read data has been submitted to the NCBI-NIH SRA (Accession #SRX351981, SRX351982, SRX351983, SRX351984, SRX351985, SRX351986, SRX351987, SRX351988, SRX351989, SRX351990, RX351991, SRX351992; BioProject ID: PRJNA218846). Reads from each pool were subsequently aligned to the reference gene sequence using Bowtie [27] allowing a maximum of two mismatches for each read pair. Alignment output files were converted to mpileup format using SAMtools [28] and mutation frequencies at each base were calculated from reads with >20 Phred base quality scores. Mutation frequencies for each pool were plotted against each base in an individual amplicon and potential mutants were identified on the basis of low frequency mutations (~1% frequency expected) occurring in each of three pools carrying the same individual line. The presence of true mutations in three pools could be readily distinguished from the presence of naturally occurring variation derived from closely related homeologous copies of the gene since this latter variation is represented in all 12 pools. 

In order to further verify the presence of candidate mutations *p*-values were calculated using Student’s t-test for whether the mutation rate of an individual pooled sample at a base position was significantly greater than the background noise attributed to sequencing error. An identified SNP was then defined as an EMS mutation if the three overlapped pools all yielded a significant *p*-value (<=0.05) (Table S1). The identification of the precise line from the set of six lines resolved by the pooling strategy was established by subsequent Sanger sequencing (BigDye v3) of the amplicon derived separately from each of the lines on an Applied Biosystems 3730xl capillary instrument (Life Technologies Inc., Grand Island, NY, USA) [27,28].

## Results

### Mutagenesis

Several populations of *B. napus* were grown from batches of 1,000 seeds mutagenized at different concentrations of EMS in an attempt to optimise mutation density. The range of mutagen concentrations used is shown in Table 1. There was no direct correlation between the frequency of induced mutations and EMS dose because the percentage survival varied considerably from batch to batch depending on environmental factors affecting growth, such as temperature, humidity and pathogen infections as well as toxicity from the mutagen (Table 1). A survival of approximately 60 % gave the best results in terms of the number of mutations identified per plant in the population.

**Table 1:** Summary of *B. napus* EMS-mutagenised populations grown for TILLING.

**EMS Dose **	**Number of seed planted **	**Survived **	**Lines carrying mutations in genes screened**
0.10%	90	36%	4.4%
0.10%	185	26%	3.2%
0.15%	108	38%	7.4%
0.15%	254	21%	3.9%
0.20%	156	19%	7.0%
0.20%	187	52%	12.8%
0.20%	222	9%	3.6%
0.20%	727	72%	not screened
0.25%	188	16%	9.6%
0.25%	874	64%	23.6%
0.25%	972	87%	15.2%
0.30%	162	10%	1.9%
0.30%	504	46%	7.7%
0.30%	828	49%	not screened
0.40%	216	5%	1.0%
0.40%	558	50%	not screened
0.40%	654	34%	not screened
0.45%	688	58%	not screened

### Population construction

A population of mutagenised *B. napus* (cv *DH12075*) M_2_ lines was constructed with each line representing a single descendant of the mutagenised M_1_ seed. A total of 3,828 lines grew to maturity (Table 1) and produced progeny. Mutant lines that produced less than 100 M_3_ seeds were not analysed further, leaving 3,158 lines in our population that produced 100 seeds or more. DNA was extracted from leaf tissue from 1,978 of these lines, arrayed as individual lines and subsequently pooled 4-fold for PCR analysis and TILLING. 

### Identification of mutations

DNA from up to 1,920 lines was screened for mutations in 26 genes using TILLING and 432 unique mutations were identified (Table 2). SNPs that were observed in multiple samples were labelled as polymorphisms in the population and were not counted as new mutations. We originally designed primers for 27 loci, but for one locus we were not able to amplify a single homeologous gene before the end of the project timeline. The data from this gene have not been included in this study because they have not been confirmed through further analysis. The total amount of DNA screened for the 26 amplicons reported in this paper was approximately 38,500 kb, indicating an average mutation density in our population of approximately 1 mutation per 109 kb, or almost 10,400 mutations per genome (based on an estimated genome size of 1,132Mb [29]). Of these, 428 were G/C to A/T transitions, two were A/T to G/C transitions, one was an A/T to C/G transversions, and one was a 3 bp insertion. This frequency of 99% G/C to A/T transitions is consistent with the mode of action of EMS [30] and similar to that reported in Arabidopsis [1], corn [31], and wheat [32]. Three hundred and seventeen of the mutant lines we identified in this project were heterozygous and 115 were homozgous for the mutations identified.

**Table 2:** Number of mutations identified and number of lines screened for each locus.

**Amplicon**	**Gene**	**Number of Mutations**	**Lines Screened**	**Amplicon Size (bp)**	Mutation Density*
bn1	Stearoyl-acyl carrier protein desaturase	21	1152	1505	1/72
bn2	Acyl-ACP thioesterase A	8	1152	1534	1/192
bn3	β-ketoacyl-ACP synthetase II	28	1920	1579	1/95
bn4	Lycopene epsilon cyclase	22	1536	1813	1/112
bn5	Acyl-ACP thioesterase B	20	1152	1655	1/84
bn6	Dihydroflavonol reductase	13	1536	1596	1/165
bn7	*GLABRA 2* homologue	21	1152	1748	1/85
bn9	Phosphatidylinositol-dependent phospholipase 2	26	1536	1377	1/70
bn13	Lysophosphatidylcholine acyltransferase	11	1152	1689	1/156
bn14	Lysophosphatidylcholine acyltransferase	21	1536	2100	1/139
bn15	*TRANSPARENT TESTA 16* homologue	17	1920	1605	1/159
bn17	Myo-inositol phosphate synthase	38	1536	1583	1/56
bn19	*TRANSPARENT TESTA 16* homologue	12	1152	1695	1/143
bn20	*DE-ETIOLATED* homologue	9	1152	1702	1/192
bn21	Diglyceride acyltransferase 1	10	1536	1181	1/150
bn22	Diglyceride acyltransferase 1	2	384	1805	1/308
bn23	Diglyceride acyltransferase 1	29	1536	1798	1/85
bn25	*BREVIPEDICELLUS* homologue	10	1152	1306	1/127
bn26	*BREVIPEDICELLUS* homologue	12	1152	1739	1/148
bn27	*MUCILAGE MODIFIED 4* homologue	7	1152	1761	1/257
bn29	Acyl-ACP thioesterase A	17	1152	1487	1/87
bn30	Acyl-ACP thioesterase B	14	1152	1392	1/98
bn31	β-ketoacyl-ACP synthetase II	17	768	1457	1/57
bn36	*FUSCA 3* homologue	15	1536	1781	1/162
bn37	*WRINKLED 1* homologue	12	768	1782	1/101
bn39	*HIGH-LEVEL EXPRESSION OF SUGAR-INDUCIBLE GENE 2* homologue	20	1536	1391	1/91
**Average:**		**17**	**1285**	**1618**	**1/109**

^*^ Total number of mutations divided by the total number of kb of DNA screened. The amplicon size used for this calculation was reduced by 200bp as the ends of the amplicons cannot be screened using the LI-COR detection system

The amplicon regions we screened varied from 1,181 to 2,100 bp with an average length of 1618 bp (Table 2). The mutations identified appear to be randomly distributed within the regions screened except for the fact that we did not detect any mutations within the first 74 bp or within the last 77 bp of the amplicon. This is consistent with previous data showing that mutations are difficult to detect within 100 bp of the primers used for PCR using the LI-COR detection system [24]. For this reason, calculations of mutation density excluded 100 bp from either end of the amplicon. The frequency of mutations varied from gene to gene (Table 2) and was correlated with the robustness of PCR amplification (i.e. how specific the primers were for that homeologue and how well the primers worked to amplify the region of interest). The number of mutations identified per gene varied from 2 to 38 with an average of 17. Mutant lines and sequences for the mutations are available on our website: http://www3.botany.ubc.ca/can-till/CanolaData.html.

### Distribution of mutations in the population

A total of 343 lines were shown to carry at least one unique mutation in the regions screened. Sixty four were found to carry mutations in more than one gene, or to carry more than one mutation in the region screened in this study. Of these, we identified two mutations in 58 of the lines, three mutations in 5 lines and four mutations in one line in the regions we screened (Table 3).

**Table 3:** Number of mutations per plant in regions screened.

**Number of M_2_ plant lines**	**Number of mutations detected per line**
1,467	0
279	1
58	2
5	3
1	4

The putative effects of the mutations were characterised using PARSESNP, a web-based programme that analyses the predicted effect of the mutation on the gene product [26], see Table 4. Just over four percent of mutations resulted in a predicted splice junction mutation or created a premature stop codon. Almost 42 percent of mutations are predicted to produce missense mutations that alter an amino acid within the predicted protein sequence, and the remainder of the mutations are predicted to be silent, either because they affect the third bp of a codon that does not alter the amino acid encoded by that triplet of bases, or because they fall in regions of the gene that are non-coding (introns or untranslated regions upstream or downstream of the translated sequence). This ratio is very similar to the ratio of 5 nonsense : 45 missense : 50 silent mutations seen in the Arabidopsis TILLING study [1].

**Table 4:** Predicted effect of mutations on gene product.

**Type of mutation **	**Number of mutations of this type**	**Frequency of mutations of this type**
nonsense	18	4.2%
missense	181	41.9%
silent	233	53.9%

### Illumina sequencing of candidate amplicons

**Figure 1:**
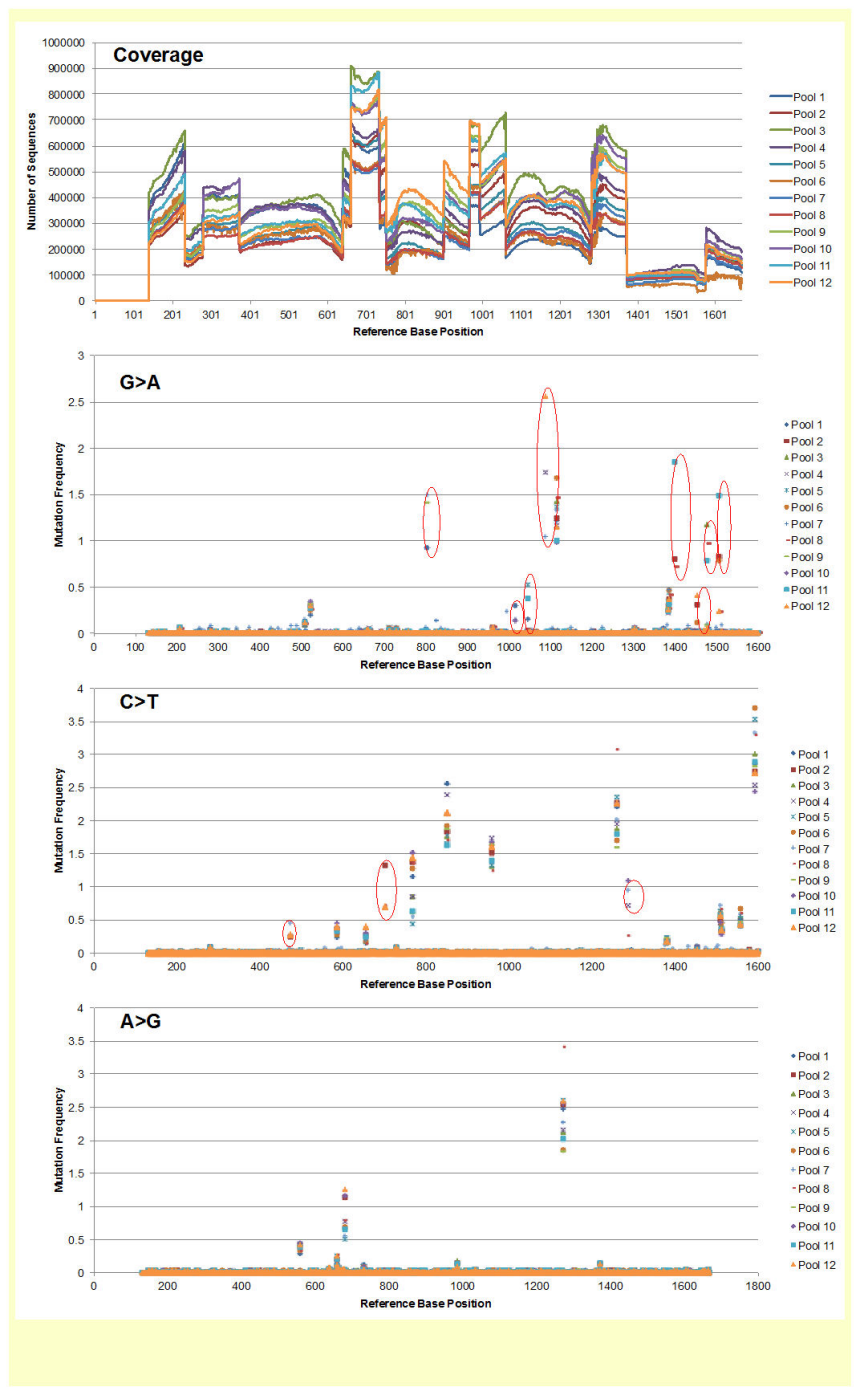
Sequencing read depth and mutation frequencies identified in 384 lines of the *B. napus* population. The top panel represents the coverage of Illumina read depth (vertical axis) across the four amplicons developed for the target gene (bn1); a total of 1,530 bp (horizontal axis). The second, third and bottom panels represent the frequency (vertical axis) of candidate mutations (C to T, G to A, and A to G, respectively) identified in three pools (circled in red) of the 12 pools used in the analysis across the length of the target region in base pairs (horizontal axis). No A to G mutations were identified in the target locus and this is consistent with the mechanism of mutation induction by EMS.

A study was carried out to assess the utility and cost-effectiveness of the Illumina sequencing platform for the identification of gene specific mutations in the mutagenized population. The approach used involved multi-dimensional pooling of extracted genomic DNA from a portion of the mutant population to screen for mutations in a target gene (BnSAD) as explained in Materials and Methods. The total sequencing data produced in this trial was able to provide very high coverage and read depth across the target region, ranging from 80,000 to 900,000 reads. The large range in depth likely reflects the fact that multiple amplicons were developed for the region and the efficiency of the paired-end Illumina sequencing for the different amplicons. The resulting depth was significantly higher than that required for routine application, however, the depth allowed a very robust evaluation of the NGS technology and enabled a more accurate extrapolation of cost effectiveness of the technique. The lower frequency of EMS mutations in specific sets of three pools that carry those lines with a mutation in a target gene is easily distinguishable from the generally higher frequency of SNPs representing differences between two homeologous loci that exist in all pools or the very low frequency of sequencing errors (Figure 1). 

**Figure 2:**
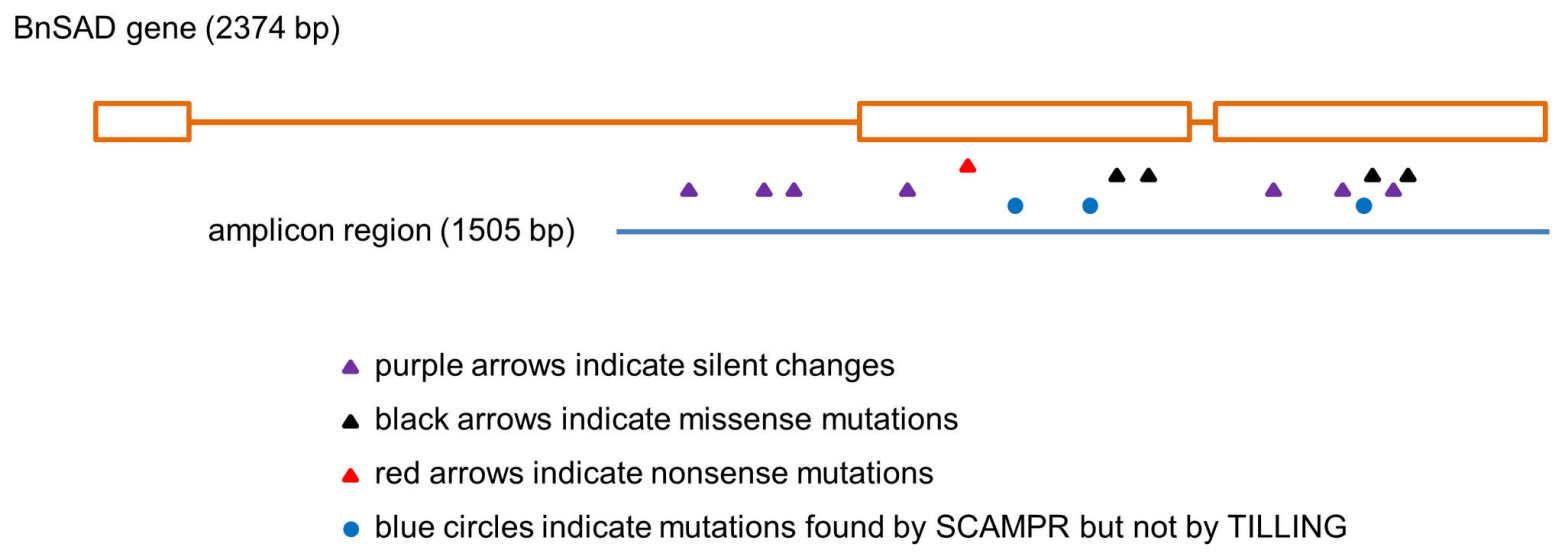
Diagrammatic representation of the distribution of mutations identified by classical TILLING and by SCAMPRing. The BnSAD gene was used as a test to compare the effectiveness of NGS sequencing (SCAMPRing) vs. classical TILLING. Purple arrows indicate silent changes in the gene that are predicted to have no effect on the protein gene product. Black arrows indicate predicted missense mutations that alter the amino acid sequence of the protein product. Red arrows indicate predicted nonsense mutations that result in premature truncation of the protein. Circles indicate mutations that were detected using SCAMPRing but not originally found with classical TILLING.

The original genotype (*DH12075*) used for the development of the mutagenized populations is double haploid and, consequently, no heterozygosity should be observed. All the identified base changes represent the expected mutations from the utilization of EMS as a mutagen (G/C to A/T transitions). A total of 11 candidate mutations were identified from the sequence data derived from various combinations of three pools representing row, column and plate specific set of lines. Candidate mutations were also supported by t-test results where base positions in all three overlap pools had significant mutation rates (*p*<=0.05; see Table S1; candidates highlighted). We were able to validate the eight mutations previously characterized by TILLING in the test locus that we sequenced (BnSAD), and we also identified three additional mutations not previously detected (Table 5 and Figure 2). These new mutations were validated by Sanger sequencing. This approach combined with increases in sequencing throughput and reduction in costs may be utilized to detect mutations in larger targeted regions or indeed whole genomes or exomes for the entire EMS mutant population to create a valuable long-term resource.

**Table 5:** Mutations identified by trial NGS sequencing (SCAMPRing) approach in 384 lines of the *B. napus* population.

**Reference Position**	**Mutation**	**Positive Indexed Pools**	**Wells To Validate**	**Sanger Sequence Validation**	**Previously Characterized**
475	C>T	2, 7, 12	DNA Box 4 - E4, E5, E6, F4, F5, F6	Confirmed	Yes
701	C>T	2, 8, 12	DNA Box 4 - G4, G5, G6, H4, H5, H6	Confirmed	Yes
803	G>A	1 , 7 , 9	DNA Box 1 - E1, E2, E3, F1, F2, F3	Confirmed	No
1017	G>A	1, 7, 10	DNA Box 2- E1, E2, E3, F1, F2, F3	Confirmed	No
1044	G>A	1, 5, 11	DNA Box 3- A1, A2, A3, B1, B2, B3	Confirmed	Yes
1089	G>A	4, 7, 12	DNA Box 4 - E10, E11, E12, F10, F11, F12	Confirmed	Yes
1288	C>T	4, 7, 10	DNA Box 2- E10, E11, E12, F10, F11, F12	Confirmed	Yes
1399	G>A	2, 8, 11	DNA Box 3 - G4, G5, G6, H4, H5, H6	Confirmed	No
1454	G>A	2, 6, 12	DNA Box 4 - C4, C5, C6, D4, D5, D6	Confirmed	Yes
1477	G>A	3, 8, 11	DNA Box 3 -A7, A8, A9, B7, B8, B9	Confirmed	Yes
1506	G>A	2, 6, 11	DNA Box 3 - C4, C5, C6, D4, D5, D6	Confirmed	Yes

## Discussion

TILLING is a reverse genetic technology that can be used to create useful genetic variability required for plant breeding programs without involving transgenic modification. Many genetic improvements in the oilseed crop canola have been based on studies in the model organism, Arabidopsis, and involve transgenic technology, but TILLING enables the development of new canola varieties using endogenous canola genes. This research can be applied directly to crop plants, saving time and money and making the research more directly relevant to growers and producers. In addition, because TILLING involves no transgenic technology, canola varieties created by TILLING are not classified as GMO by the European Union and this allows the expansion of these varieties to that market, thus providing economic advantages to producers.

We have generated an EMS-mutagenised population of 3,158 individual lines of the *Brassica napus* cultivar *DH12075* and screened for mutations in 1,920 lines. A total of 432 unique mutations in 26 genes were identified by TILLING, thus providing multiple mutant alleles for each gene. The genes we screened were chosen based on requests from researchers studying seed properties in *Brassica napus* and are listed in Table 2. Sequence data for specific mutations and seed lines are available online at: http://www3.botany.ubc.ca/can-till/CanolaData.html. Almost 42 percent of the mutations we identified were missense alleles resulting in a change in one of the amino acids in the protein product of the gene. Just over four percent were nonsense alleles resulting from the insertion of a premature stop codon into the coding region of the gene, or in the elimination of a conserved splice junction site. The remainder of the alleles we identified are predicted to be silent, either because they are in non-coding DNA or because they affect the third bp of a codon which does not change the amino acid encoded by that codon. 

In our population, there was considerable gene-to-gene variation in mutation density, ranging from 1/56 kb to 1/308 kb. It is important to keep in mind that identification of mutations using the classical TILLING technique is dependent on both robust PCR amplification of a single gene product, as well as efficient digestion with the CJE enzyme. We suggest that these factors likely affect the mutation density more than other factors such as gene location or amplicon size. The highest mutation densities we saw in this study were for bn17 (1/56 kb) and bn31 (1/57 kb), and for both of these amplicons the primers used for PCR amplification worked well and appeared to amplify a single PCR product on the gel (see Figure S3). The lowest mutation frequencies we observed were for bn22 (1/308 kb) and bn27 (1/257 kb). Amplification was poor for both of these amplicons (see Figure S4) and, in fact, in all cases where the mutation density was below 1/140 kb, the PCR primers used for those genes amplified poorly, so we expect that mutations were missed in some samples. 

An additional complication in designing primers in polyploid species such as *B. napus* is the consideration of amplification of homeologous genes. If primers must be designed to specifically amplify one of the two (or more) homeologous pairs of genes this is not always straightforward in the absence of sequenced genomes for many crop species. Pre-testing of primers in order to find ones that amplify only a single target gene is necessary in this case, although we and others have also found that this was necessary with some diploids with large genomes such as corn [31] and poplar [33]. If PCR primers amplify more than one homeologous or paralogous gene this complicates the analysis of the results from TILLING because further work must be done in order to determine which of the homologues carries the mutation identified. Polymorphisms between the different genes are easily distinguished from bona fide mutations because they will be present in all of the samples rather than in just one. In our project re-designing of primers to amplify one homeologue was necessary for most loci. It is important to note that, however, that of the 27 requests we received, we were able to identify mutations in 26 of these loci, without employing any additional strategies to differentiate between homeologous genes. The initial primers we designed for the remaining gene appeared to amplify both homeologues and we did not have time to re-design primers for this gene within the time frame of this project. 

A number of strategies have been developed to address the problem of sequence similarity between homeologous, or paralogous genes. These include the use of restriction endonuclease sites to differentiate between the two loci [34]. This strategy was successfully employed by Cooper et al., 2008 [34], in soybean where PCR amplification products that included more than one locus were digested with a restriction endonuclease that only recognised one of the homeologous loci before mutation detection. Another strategy used to overcome the problem of gene duplication is nested PCR to specifically amplify one locus or another. This is an old reverse genetics strategy that has recently been demonstrated in the peanut TILLING project by Knoll et al., 2011 [35]. In this case, the initial round of PCR uses primers that amplify both homeologous genes. This is followed by a second round of PCR using primers that amplify only one of the homeologous loci. On the other hand, gene redundancy can be used as an advantage in some cases because it allows for screening of mutations in multiple homeologous genes at the same time, followed by further analysis to determine which of the homeologues carries the identified mutations of interest [8]. 

High mutation densities of between 1/15 kb and 1/50 kb have been reported for some polyploid populations in different species [21,23,32,36,37]. However, the number of genes TILLed in these studies is low, and recent TILLING projects in polyploid plants such as tobacco, peanut and tef have reported mutation densities of 1/1423 kb, 1/1000 kb and 1/243 kb, respectively [14,35,38]. On the other hand, a diploid Arabidopsis *Landsberg erecta* population has been reported to carry an estimated mutation density of 1/89 kb [39], and a diploid *B. rapa* population described by Stephenson et al.[22] is reported to have a mutation density of 1/60 kb. Overall, frequencies in polyploid TILLING populations generated using EMS range from 1/15 kb for one population of *B. napus* [23] to 1/1423 kb for a *Nicotiana tabacum* population [38]. This compares to frequencies for diploid populations of 1/60 kb (*B. rapa*) [22] to 1/737 kb (*Solanum lycopersicum*) [9]. While it is possible that, in general, polyploids are capable of surviving higher mutation densities than diploids, the higher density seen in some diploid studies and the lower mutation density seen in some polyploid populations may simply reflect differences in mutagenesis or in screening rather than ploidy *per se*. In the *Landsberg erecta* population, for example, a higher mutation density was achieved by allowing the selection of plants with very low fertility [34]. In our population, as well as in TILLING populations in Arabidopsis and soybean [34], variation in survival and in mutation density was observed from batch to batch, even using identical concentrations of EMS and identical batches of seeds as starting material for mutagenesis (Table 1). For this project, we did not screen any plant that gave less than 100 seeds, thereby potentially eliminating the most heavily mutagenised plant genomes in our population. In addition, primers for some genes did not work well consistently and thus we may have missed mutation in those genes as mentioned above. Our justification for stringent selection of fertile plants was based on the fact that we only wanted to isolate mutations from plants that had produced enough seeds to be of value to researchers requesting mutant lines carrying mutations in their genes of interest. Another advantage of generating plants that are not too heavily mutagenized is the fact that analysis of the function of the mutant gene of interest is easier in plants where the background mutation level is not so high that multiple mutations complicate analysis of the phenotype observed. This may explain the lower mutation density we observed relative to other Brassica populations [21,22], although it may also be that if more genes were screened in those populations the average mutation density would decrease. 

In reality, the different studies of EMS-mutagenised populations are so variable, in terms of the numbers of plants screened and the numbers of genes tested that a valid comparison may only be possible through the use of genome sequencing to determine the precise mutation density in each plant in each population. It has been suggested that polyploid species, in contrast to diploids, can withstand higher mutation densities because of genetic redundancy and this seems like an intuitively rational concept. However, given the small sample sizes for most TILLING studies, and the differences in mutagenesis and population construction, the data available so far are not sufficient to determine the absolute effect of ploidy on the mutation load that can be tolerated.

The average mutation density in our population was approximately 1/109 kb (one mutation for every 109 kb of DNA screened). As far as we are aware, this is the largest number of genes screened in one population for any polyploid plant species and, therefore, we believe that our results provide an accurate picture of the overall mutation density in the population. To the best of our knowledge, no more than six genes have ever been screened using TILLING in any other polyploid population, and the average number of genes screened in other studies is 3.5. We observed considerable variation from gene to gene in the 26 genes we screened in this study. If we had selected only three of those genes, we would have observed an average mutation density somewhere in between 1/60 kb and 1/243 kb (depending on which three genes we had chosen). For this reason, we believe that the average mutation density reported here is significantly more robust than the average mutation rates reported previously where only a small number of genes have been targetted. 

In order to determine whether direct sequencing using NGS technology (SCAMPRing) was a more efficient way to identify mutations in our *B. napus* population, we used amplicon sequencing of multiple samples simultaneously and found those results comparable to the results observed using the classical TILLING technique (PCR amplification followed by enzymatic digestion and acrylamide gel detection). However, the approach did identify additional mutations in the target gene that were not identified by TILLING. The strategy described here using Illumina sequencing provided a high depth of coverage and enabled a very robust evaluation of the technology for this application. The amplification and sequencing of individual amplicons from the population was more labour-intensive and more expensive than TILLING. In order for this NGS strategy to be more economical and used routinely, multiple aspects need to be addressed. Firstly, the overall depth of sequencing generated for this trial was very high and could be reduced greatly (at least 100 fold) yet still provide an equivalent efficiency in calling candidate mutations. Secondly, the GAIIx platform used for the sequencing has now been supplanted by the HiSeq2000 platform (Illumina Inc.) that provides approximately eight fold more data per lane than the GAIIx for approximately the same amount of consumables. The recent availability of additional library indexes/barcodes in commercial 96-well plate based kits (e.g. TruSeq 96, Illumina Inc.) or custom kits (GBS 384, [40]) provide a much reduced labour requirement for library construction and a pooling depth that enables true multidimensional pooling, thus enabling resolution of mutations to individual lines within the population. Based on the above we calculate that it would be possible to screen up to 10 different 2 kb amplicons at a depth of ~100 fold per allele in 3,072 mutant lines in a total of 116 barcoded tri-dimensional row, column and plates pools in a single lane on the HiSeq platform and at a plexity no higher than 96 lines (192 alleles) in any one pool. As described previously by Tsai et al. 2011 [13] this latter point of limiting the number line per pool is important since a single allele mutation in one diploid individual in a pool containing 96 lines (1 in 192 alleles) gives an approximately 0.5% signal. This is significantly higher than overall noise due to sequencing error, normally <0.05% based on current Illumina data qualities, but higher levels of plexity could begin to compromise the effectiveness of the strategy. Looking to the future, new streamlined methodologies for barcoding of samples during PCR amplification rather than post-amplification, such as described by Wells et al. 2013 [15], will introduce both technical and cost efficiencies to the process of producing NGS libraries for this application. As well, relatively new platforms that enable automated high-complexity PCR amplification of many target regions in multiple lines (e.g. Access Array, Fluidigm Inc., San Francisco, CA, USA) or the capture of entire exomes (SeqCap EZ, Roche Nimblegen Inc., Basel, Switzerland) can provide an increased range in the amount of sequence that is surveyed together with low labour requirements. Taken together these developments would enable SCAMPRing on massive scale in entire mutagenized populations for significantly reduced costs. 

Given that there are close to 9,000 mutations in each genome in the *B. napus* population described here, and that these mutations will be distributed across the genome in potentially critical positions impacting gene expression, it would be ideal to deduce the entire complement of mutations in every line of the population. Since a reference genome for *B. napus* will be available shortly (I. Parkin and A. Sharpe, unpublished) the most thorough approach for the detection of mutations would be to sequence the entire genome of each plant in the population. Future technological advancements in sequencing could allow such an approach to be undertaken cost effectively. Until such time the various NGS strategies are well proven in polyploid genome like *B. napus* and until sequencing costs decrease further, TILLING will remain a method of choice for detection of point mutations.

An important step in the development of value-added canola for researchers and producers is the identification of genetic variation that can be used in breeding programs. The mutagenised *B. napus* population produced in this project represents a valuable source of such genetic variability that should be useful for scientists and breeders to investigate and improve many aspects of canola biology for years to come. Indeed our TILLING of this population has already shown that we can identify lines with mutations in genes that may be involved in important agronomic traits including seed oil biosynthesis, carotenoid production, and meristem development. This is an important step in the development of value-added canola crop varieties.

## Conclusion

The populations generated for TILLING in any species provide valuable resources for teaching and research. We have generated a *B. napus* population of more than 3,000 lines and have shown that TILLING is an effective way to identify mutations in this species in spite of the redundant nature of its polyploid genome. We have identified 432 unique mutations in 26 different genes, reflecting a mutation density of one per 109 kb. We have also successfully verified the utility of NGS technologies as a potentially powerful and routine approach for the identification of mutations in polyploid species of this nature. Different reverse genetics techniques in plants have their advantages and disadvantages depending on the questions being asked. Chemical mutagenesis and TILLING is an important contribution to this arsenal of mutations in *B. napus* genes. The genes that we have identified through TILLING can now be used for classical genetic analysis that will lead to a better understanding of their function(s) and their interactions with other genes in this important crop species. Most of the mutant lines described in this paper are publically available by contacting the authors and a list of sequenced mutations is available on our website: http://www3.botany.ubc.ca/can-till/CanolaData.html.

## Supporting Information

Figure S1 **Schematic representation of SCAMPRing.** Sequence Candidate Amplicons in Multiple Parallel Reactions (SCAMPRing) employs next generation sequencing to identify mutations in a target gene, BnSAD. Pooling and sequencing strategies are shown in this figure. A total of four amplicons were generated for the gene and then amplified in 384 mutant lines in a total of 12 row, column and plate pools (see Fig S2 for exact description of pooling design). The pools were indexed using unique Illumina barcodes for library construction and sequencing on a GAIIx platform.
(DOCX)

Figure S2 **Schematic representation of the pooling design employed for amplicon generation in a target gene, BnSAD.** A total of 384 lines were used for PCR amplification with products from 96 being pooled and used for Illumina library construction with a unique barcoded adapter. A total of 12 row, column and plate pools were generated such that each line was amplified in three different pools to enable the identification of a mutation in a set of six lines. The different pools are represented by different colours in each well of the four 96 well plates containing the 384 different lines.
(DOCX)

Figure S3 **LI-COR TILLING gel using the bn17 primer set.** Amplification is robust and produces lots of product. Samples can be observed and scored in almost every lane. Images were annotated using GelBuddy [25] to demark the 96 lanes in both the 700 nm and 800 nm channel images of the 96-well gel.
(DOCX)

Figure S4 **LI-COR TILLING gel using the bn27 primer set.** Amplification is variable and produces more product in some lanes than in others. In some lanes no sample can be observed and thus we are unable to discern whether or not there is a mutation in that pool. Images were annotated using GelBuddy [25] to demark the 96 lanes in both the 700 nm and 800 nm channel images of the 96-well gel.
(DOCX)

Table S1 **Raw data from EMS mutation candidates found using Illumina sequencing.**
(XLSX)

## References

[1] Greene EA, Codomo CA, Taylor NE, Henikoff JG, Till BJ et al. (2003) Spectrum of chemically induced mutations from a large-scale reverse-genetic screen in Arabidopsis. Genetics 164: 731–740. PubMed: 12807792. 12807792 10.1093/genetics/164.2.731 PMC1462604

[2] Bleecker AB, Kende H (2000) Ethylene: a gaseous signal molecule in plants. Annu Rev Cell Dev Biol 16: 1–18. doi:10.1146/annurev.cellbio.16.1.1. PubMed: 11031228. 11031228

[3] Byrne ME (2006) Shoot meristem function and leaf polarity: the role of class III HD-ZIP genes. PLoS Genet 2: e89 Accessed 27 November 2012 16846251 10.1371/journal.pgen.0020089 PMC1484593

[4] Eckardt N (2007) Positive and Negative Feedback Coordinate Regulation of Disease Resistance Gene Expression. Plant Cell Online 19: 2700–2702. Accessed 14 December 2012

[5] Colbert T, Till BJ, Tompa R, Reynolds S, Steine MN et al. (2001) High-throughput screening for induced point mutations. Plant Physiol 126: 480–484. doi:10.1104/pp.126.2.480. PubMed: 11402178. 11402178 PMC1540114

[6] McCallum CM, Comai L, Greene Ea, Henikoff S (2000) Targeted screening for induced mutations. Nat Biotechnol 18: 455–457. doi:10.1038/74542. PubMed: 10748531. 10748531

[7] Raghavan C, Naredo MEB, Wang H, Atienza G, Liu B, et al. (2006) Rapid method for detecting SNPs on agarose gels and its application in candidate gene mapping. Mol Breed 19: 87–101. Accessed 26 October 2012

[8] Dong C, Vincent K, Sharp P (2009) Simultaneous mutation detection of three homoeologous genes in wheat by High Resolution Melting analysis and Mutation Surveyor. BMC Plant Biol 9: 143 Accessed 12 August 2013 19958559 10.1186/1471-2229-9-143 PMC2794869

[9] Gady AL, Hermans FW, Van de Wal MH, van Loo EN, Visser RG, et al. (2009) Implementation of two high through-put techniques in a novel application: detecting point mutations in large EMS mutated plant populations. Plant Methods 5: 13 Accessed 22 August 2013 19811648 10.1186/1746-4811-5-13 PMC2763861

[10] Wang TL, Uauy C, Robson F, Till B (2012) TILLING in extremis. Plant Biotechnol J 10: 761–772. Accessed 22 March 2013 22651686 10.1111/j.1467-7652.2012.00708.x

[11] Rigola D, van Oeveren J, Janssen A, Bonné A, Schneiders H, et al. (2009) High-throughput detection of induced mutations and natural variation using KeyPoint technology. PLoS One 4: e4761 Accessed 6 November 2012 19283079 10.1371/journal.pone.0004761 PMC2654077

[12] Marroni F, Pinosio S, Di Centa E, Jurman I, Boerjan W, et al. (2011) Large-scale detection of rare variants via pooled multiplexed next-generation sequencing: towards next-generation Ecotilling. Plant J 67: 736–745. Accessed 22 August 2013 21554453 10.1111/j.1365-313X.2011.04627.x

[13] Tsai H, Howell T, Nitcher R, Missirian V, Watson B, et al. (2011) Discovery of rare mutations in populations: TILLING by sequencing. Plant Physiol 156: 1257–1268. Accessed 29 October 2012 21531898 10.1104/pp.110.169748 PMC3135940

[14] Zhu Q, Smith SM, Ayele M, Yang L, Jogi A, et al. (2012) High-throughput discovery of mutations in tef semi-dwarfing genes by next-generation sequencing analysis. Genetics 192: 819–829. Accessed 2 September 2013 22904035 10.1534/genetics.112.144436 PMC3522160

[15] Wells R, Trick M, Fraser F, Soumpourou E, Clissold L, et al. (2013) Sequencing-based variant detection in the polyploid crop oilseed rape. BMC Plant Biol 13: 111 Accessed 12 August 2013 23915099 10.1186/1471-2229-13-111 PMC3750413

[16] Mamanova L, Coffey AJ, Scott CE, Kozarewa I, Turner EH et al. (2010) Target-enrichment strategies for next-generation sequencing. Nat Methods 7: 111–118. doi:10.1038/NMETH.1419. PubMed: 20111037. 20111037

[17] Bus A, Hecht J, Huettel B, Reinhardt R, Stich B (2012) High-throughput polymorphism detection and genotyping in Brassica napus using next-generation RAD sequencing. BMC Genomics 13: 281. doi:10.1186/1471-2164-13-281. PubMed: 22726880. 22726880 PMC3442993

[18] Stefansson BR, Kondra ZP (1975) Tower summer rape. Can J Plant Sci 55: 343–344. doi:10.4141/cjps75-053.

[19] Johnson GH, Keast DR, Kris-Etherton PM (2007) Dietary modeling shows that the substitution of canola oil for fats commonly used in the United States would increase compliance with dietary recommendations for fatty acids. J Am Diet Assoc 107: 1726–1734. Accessed 14 May 2013 17904932 10.1016/j.jada.2007.07.015

[20] Gebauer SK, Psota TL, Harris WS, Kris-Etherton PM (2006) N-3 Fatty Acid Dietary Recommendations and Food Sources To Achieve Essentiality and Cardiovascular Benefits. Am J Clin Nutr 83: 1526S–1535S. PubMed: 16841863. 16841863 10.1093/ajcn/83.6.1526S

[21] Wang N, Wang Y, Tian F, King GJ, Zhang C, et al. (2008) A functional genomics resource for Brassica napus: development of an EMS mutagenized population and discovery of FAE1 point mutations by TILLING. New Phytol 180: 751–765. Accessed 10 December 2012 18811617 10.1111/j.1469-8137.2008.02619.x

[22] Stephenson P, Baker D, Girin T, Perez A, Amoah S et al. (2010) A rich TILLING resource for studying gene function in Brassica rapa. BMC Plant Biol 10: 62. doi:10.1186/1471-2229-10-62. PubMed: 20380715. 20380715 PMC2923536

[23] Harloff H-J, Lemcke S, Mittasch J, Frolov A, Wu JG, et al. (2012) A mutation screening platform for rapeseed (Brassica napus L.) and the detection of sinapine biosynthesis mutants. Theor Appl Genet 124: 957–969. Accessed 8 August 2013 22198204 10.1007/s00122-011-1760-z

[24] Till BJ, Burtner C, Comai L, Henikoff S (2004) Mismatch cleavage by single-strand specific nucleases. Nucleic Acids Res 32: 2632–2641. Accessed 23 November 2012 15141034 10.1093/nar/gkh599 PMC419476

[25] Zerr T, Henikoff S (2005) Automated band mapping in electrophoretic gel images using background information. Nucleic Acids Res 33: 2806–2812. Accessed 6 November 2012 15894797 10.1093/nar/gki580 PMC1126905

[26] Taylor NE (2003) PARSESNP: a tool for the analysis of nucleotide polymorphisms. Nucleic Acids Res 31: 3808–3811. Accessed 10 September 2012 12824424 10.1093/nar/gkg574 PMC168980

[27] Langmead B, Trapnell C, Pop M, Salzberg SL (2009) Ultrafast and memory-efficient alignment of short DNA sequences to the human genome. Genome Biol 10: R25 Accessed 28 February 2013 19261174 10.1186/gb-2009-10-3-r25 PMC2690996

[28] Li H, Handsaker B, Wysoker A, Fennell T, Ruan J, et al. (2009) The Sequence Alignment/Map format and SAMtools. Bioinformatics 25: 2078–2079. Accessed 27 February 2013 19505943 10.1093/bioinformatics/btp352 PMC2723002

[29] Johnston JS, Pepper AE, Hall AE, Chen ZJ, Hodnett G, et al. (2005) Evolution of genome size in Brassicaceae. Ann Bot 95: 229–235. Accessed 30 January 2013 15596470 10.1093/aob/mci016 PMC1950721

[30] Burns PA, Allen FL, Glickman BW (1986) DNA SEQUENCE ANALYSIS OF MUTAGENICITY AND. Genetics 113: 811–819. PubMed: 3527868. 3527868 10.1093/genetics/113.4.811 PMC1202914

[31] Till BJ, Reynolds SH, Weil C, Springer N, Burtner C, et al. (2004) Discovery of induced point mutations in maize genes by TILLING. BMC Plant Biol 4: 12 Accessed 9 November 2012 15282033 10.1186/1471-2229-4-12 PMC512284

[32] Slade AJ, Fuerstenberg SI, Loeffler D, Steine MN, Facciotti D (2005) A reverse genetic, nontransgenic approach to wheat crop improvement by TILLING. Nat Biotechnol 23: 75–81. Accessed 26 October 2012 15580263 10.1038/nbt1043

[33] Gilchrist EJ, Haughn GW, Ying CC, Otto SP, Zhuang J, et al. (2006) Use of Ecotilling as an efficient SNP discovery tool to survey genetic variation in wild populations of Populus trichocarpa. Mol Ecol 15: 1367–1378. Accessed 2 December 2012 16626459 10.1111/j.1365-294X.2006.02885.x

[34] Cooper JL, Till BJ, Laport RG, Darlow MC, Kleffner JM, et al. (2008) TILLING to detect induced mutations in soybean. BMC Plant Biol 8: 9 Accessed 26 October 2012 18218134 10.1186/1471-2229-8-9 PMC2266751

[35] Knoll JE, Ramos ML, Zeng Y, Holbrook CC, Chow M, et al. (2011) TILLING for allergen reduction and improvement of quality traits in peanut (Arachis hypogaea L). BMC Plant Biol 11: 81. Accessed 9 September 2013 10.1186/1471-2229-11-81 PMC3113929 21569438

[36] Uauy C, Paraiso F, Colasuonno P, Tran RK, Tsai H, et al. (2009) A modified TILLING approach to detect induced mutations in tetraploid and hexaploid wheat. BMC Plant Biol 9: 115 Accessed 7 September 2013 19712486 10.1186/1471-2229-9-115 PMC2748083

[37] Chawade A, Sikora P, Bräutigam M, Larsson M, Vivekanand V et al. (2010) Development and characterization of an oat TILLING-population and identification of mutations in lignin and β-glucan biosynthesis genes. BMC Plant Biol: 86. 10.1186/1471-2229-10-86 PMC3017761 20459868

[38] Reddy TV, Dwivedi S, Sharma NK (2012) Development of TILLING by sequencing platform towards enhanced leaf yield in tobacco. Ind Crops Prod 40: 324–335. doi:10.1016/j.indcrop.2012.03.031.

[39] Martín B, Ramiro M, Martínez-Zapater JM, Alonso-Blanco C (2009) A high-density collection of EMS-induced mutations for TILLING in Landsberg erecta genetic background of Arabidopsis. BMC Plant Biol 9: 147 Accessed 6 December 2012 20003424 10.1186/1471-2229-9-147 PMC2803491

[40] Poland J, Brown PJ, Sorrells ME, Jannink J-L (2012) Development of high-density genetic maps for barley and wheat using a novel two-enzyme genotyping-by-sequencing approach. PLoS One 7: e32253 Accessed 27 February 2013 22389690 10.1371/journal.pone.0032253 PMC3289635

