# Supplementary material for: A Mutant Brassica napus (Canola) Population for the Identification of New Genetic Diversity via TILLING and Next Generation Sequencing
Source: PLoS One. 2013 Dec 20;8(12):e84303. doi: 10.1371/journal.pone.0084303 (PMC3869819; doi:10.1371/journal.pone.0084303)
Supplement: Figure S1 — Schematic representation of SCAMPRing. Sequence Candidate Amplicons in Multiple Parallel Reactions (SCAMPRing) employs next generation sequencing to identify mutations in a target gene, BnSAD. Pooling and sequencing strategies are shown in this figure. A total of four amplicons were generated for the gene and then amplified in 384 mutant lines in a total of 12 row, column and plate pools (see Fig S2 for exact description of pooling design). The pools were indexed using unique Illumina barcodes for library construction and sequencing on a GAIIx platform. (DOCX) [file pone.0084303.s001.docx]

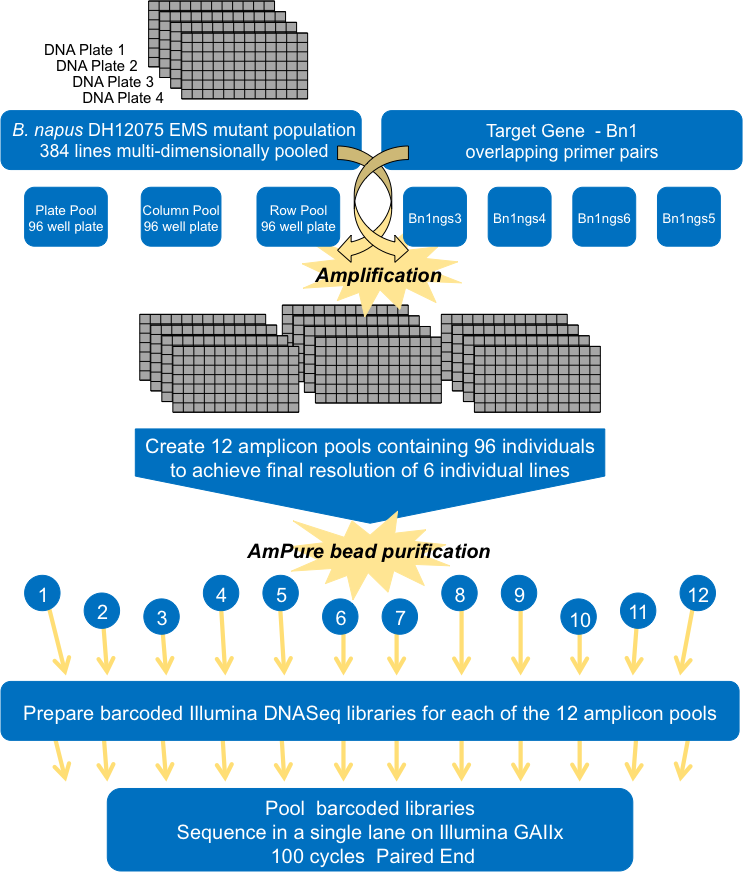


**Figure S1.** **Schematic representation of SCAMPRing**.

Sequence Candidate Amplicons in Multiple Parallel Reactions (SCAMPRing) employs next generation sequencing to identify mutations in a target gene, BnSAD. Pooling and sequencing strategies are shown in this figure. A total of four amplicons were generated for the gene and then amplified in 384 mutant lines in a total of 12 row, column and plate pools (see Supp. Fig 2 for exact description of pooling design). The pools were indexed using unique Illumina barcodes for library construction and sequencing on a GAIIx platform.
