# Supplementary material for: A Mutant Brassica napus (Canola) Population for the Identification of New Genetic Diversity via TILLING and Next Generation Sequencing
Source: PLoS One. 2013 Dec 20;8(12):e84303. doi: 10.1371/journal.pone.0084303 (PMC3869819; doi:10.1371/journal.pone.0084303)
Supplement: Figure S2 — Schematic representation of the pooling design employed for amplicon generation in a target gene, BnSAD. A total of 384 lines were used for PCR amplification with products from 96 being pooled and used for Illumina library construction with a unique barcoded adapter. A total of 12 row, column and plate pools were generated such that each line was amplified in three different pools to enable the identification of a mutation in a set of six lines. The different pools are represented by different colours in each well of the four 96 well plates containing the 384 different lines. (DOCX) [file pone.0084303.s002.docx]

**
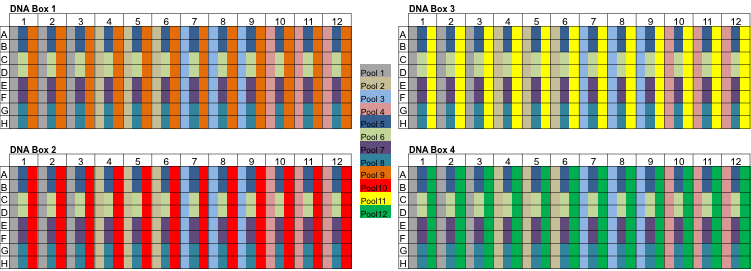
**

**Figure S2.** **Schematic representation of the pooling design employed for amplicon generation in a target gene, BnSAD**.

A total of 384 lines were used for PCR amplification with products from 96 being pooled and used for Illumina library construction with a unique barcoded adapter. A total of 12 row, column and plate pools were generated such that each line was amplified in three different pools to enable the identification of a mutation in a set of six lines. The different pools are represented by different colours in each well of the four 96 well plates containing the 384 different lines.
