# Supplementary material for: A Mutant Brassica napus (Canola) Population for the Identification of New Genetic Diversity via TILLING and Next Generation Sequencing
Source: PLoS One. 2013 Dec 20;8(12):e84303. doi: 10.1371/journal.pone.0084303 (PMC3869819; doi:10.1371/journal.pone.0084303)
Supplement: Figure S3 — LI-COR TILLING gel using the bn17 primer set. Amplification is robust and produces lots of product. Samples can be observed and scored in almost every lane. Images were annotated using GelBuddy [25] to demark the 96 lanes in both the 700 nm and 800 nm channel images of the 96-well gel. (DOCX) [file pone.0084303.s003.docx]

**
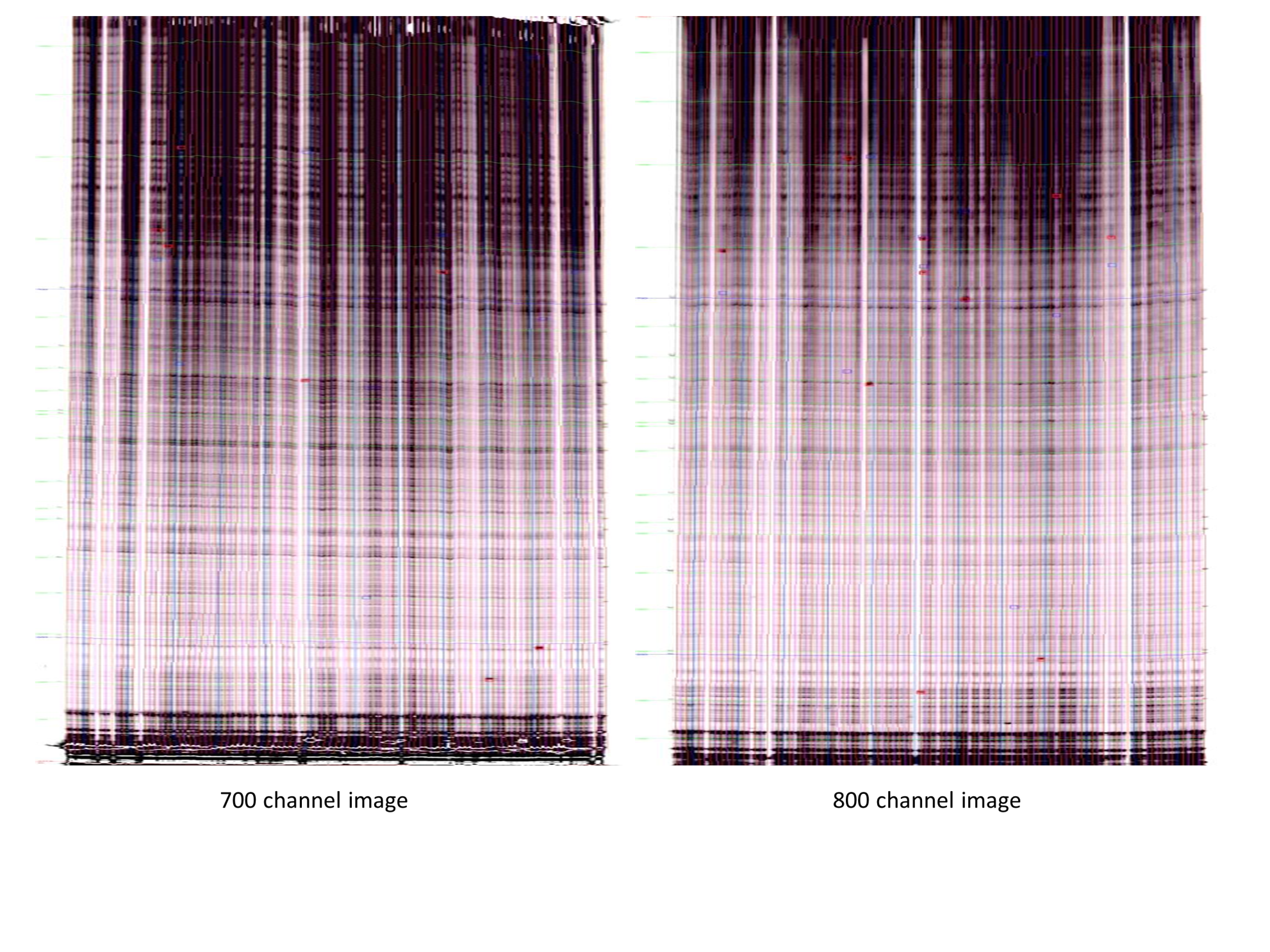
**

**Figure S3. LI-COR TILLING gel using the bn17 primer set.** Amplification is robust and produces lots of product. Samples can be observed and scored in almost every lane. Images were annotated using GelBuddy (Zerr T, Henikoff S (2005) Nucleic Acids Res 33: 2806–2812) to demark the 96 lanes in both the 700 nm and 800 nm channel images of the 96-well gel.
