# Supplementary material for: A Mutant Brassica napus (Canola) Population for the Identification of New Genetic Diversity via TILLING and Next Generation Sequencing
Source: PLoS One. 2013 Dec 20;8(12):e84303. doi: 10.1371/journal.pone.0084303 (PMC3869819; doi:10.1371/journal.pone.0084303)
Supplement: Figure S4 — LI-COR TILLING gel using the bn27 primer set. Amplification is variable and produces more product in some lanes than in others. In some lanes no sample can be observed and thus we are unable to discern whether or not there is a mutation in that pool. Images were annotated using GelBuddy [25] to demark the 96 lanes in both the 700 nm and 800 nm channel images of the 96-well gel. (DOCX) [file pone.0084303.s004.docx]

**
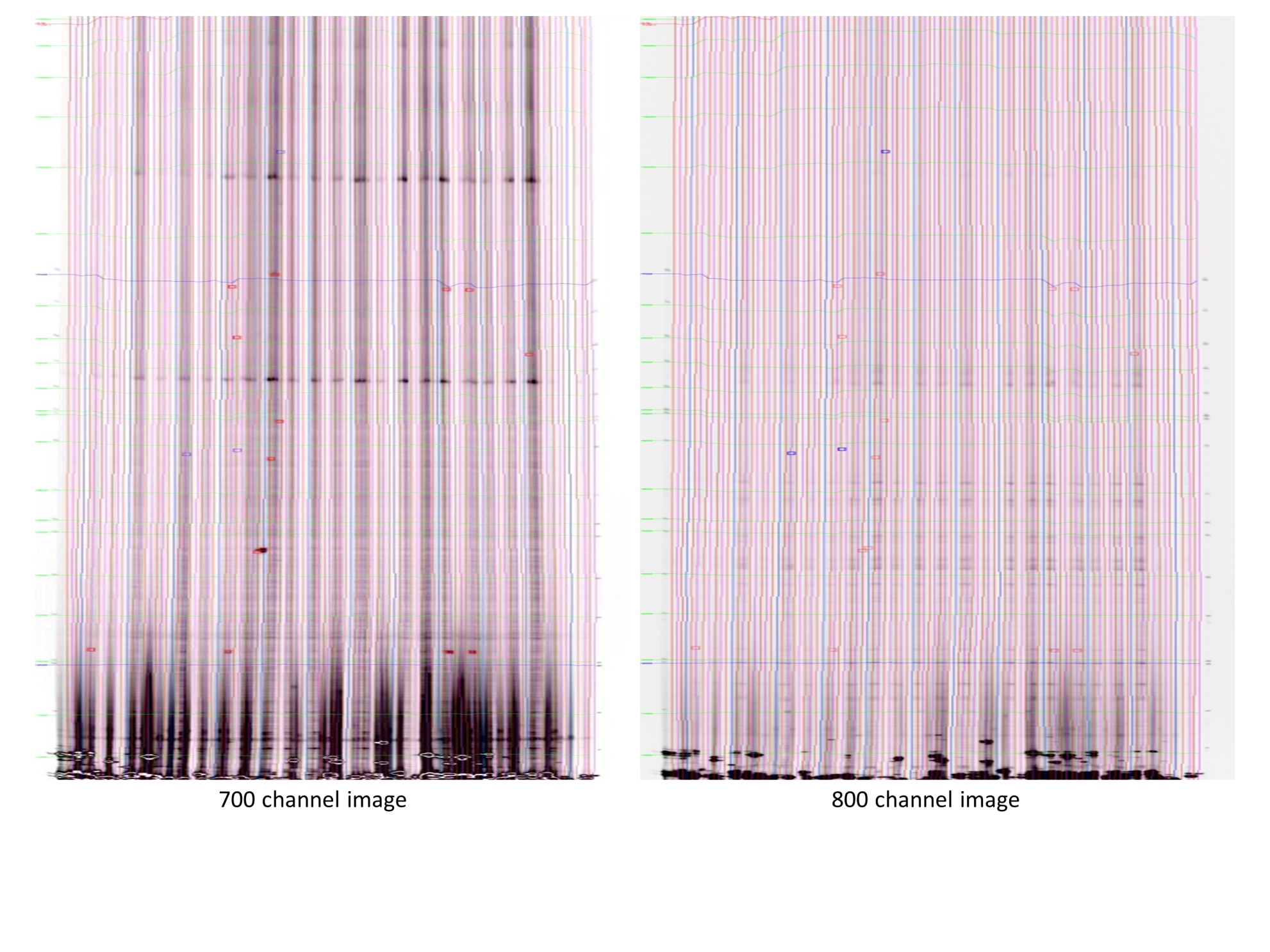
**

**Figure S4. LI-COR TILLING gel using the bn27 primer set.** Amplification is variable and produces more product in some lanes than in others. In some lanes no sample can be observed and thus we are unable to discern whether or not there is a mutation in that pool. Images were annotated using GelBuddy (Zerr T, Henikoff S (2005) Nucleic Acids Res 33: 2806–2812) to demark the 96 lanes in both the 700 nm and 800 nm channel images of the 96-well gel.
